# The Role of Type II Fatty Acid Synthesis Enzymes FabZ, ODSCI, and ODSCII in the Pathogenesis of *Toxoplasma gondii* Infection

**DOI:** 10.3389/fmicb.2021.703059

**Published:** 2021-08-31

**Authors:** Xiao-Pei Xu, Hany M. Elsheikha, Wen-Ge Liu, Zhi-Wei Zhang, Li-Xiu Sun, Qin-Li Liang, Ming-Xin Song, Xing-Quan Zhu

**Affiliations:** ^1^State Key Laboratory of Veterinary Etiological Biology, Key Laboratory of Veterinary Parasitology of Gansu Province, Lanzhou Veterinary Research Institute, Chinese Academy of Agricultural Sciences, Lanzhou, China; ^2^Heilongjiang Key Laboratory for Zoonosis, College of Veterinary Medicine, Northeast Agricultural University, Harbin, China; ^3^Faculty of Medicine and Health Sciences, School of Veterinary Medicine and Science, University of Nottingham, Nottingham, United Kingdom; ^4^College of Veterinary Medicine, Shanxi Agricultural University, Taigu, China; ^5^Key Laboratory of Veterinary Public Health of Higher Education of Yunnan Province, College of Veterinary Medicine, Yunnan Agricultural University, Kunming, China

**Keywords:** *Toxoplasma gondii*, virulence factors, FASII enzymes, FabZ, ODSCI, ODSCII

## Abstract

*Toxoplasma gondii* is an obligate intracellular protozoan parasite, which has a worldwide distribution and can infect a large number of warm-blooded animals and humans. *T. gondii* must colonize and proliferate inside the host cells in order to maintain its own survival by securing essential nutrients for the development of the newly generated tachyzoites. The type II fatty acid biosynthesis pathway (FASII) in the apicoplast is essential for the growth and survival of *T. gondii*. We investigated whether deletion of genes in the FASII pathway influences the *in vitro* growth and *in vivo* virulence of *T. gondii*. We focused on beta-hydroxyacyl-acyl carrier protein dehydratase (FabZ) and oxidoreductase, short chain dehydrogenase/reductase family proteins ODSCI and ODSCII. We constructed *T. gondii* strains deficient in *FabZ, ODSCI*, and *ODSCII* using CRISPR-Cas9 gene editing technology. The results of immunofluorescence assay, plaque assay, proliferation assay and egress assay showed that in RHΔ*FabZ* strain the apicoplast was partly lost and the growth ability of the parasite *in vitro* was significantly inhibited, while for RHΔ*ODSCI* and RHΔ*ODSCII* mutant strains no similar changes were detected. RHΔ*FabZ* exhibited reduced virulence for mice compared with RHΔ*ODSCI* and RHΔ*ODSCII*, as shown by the improved survival rate. Deletion of *FabZ* in the PRU strain significantly decreased the brain cyst burden in mice compared with PRUΔ*ODSCI* and PRUΔ*ODSCII*. Collectively, these findings suggest that FabZ contributes to the growth and virulence of *T. gondii*, while ODSCI and ODSCII do not contribute to these traits.

## Introduction

*Toxoplasma gondii* is an obligate intracellular protozoan, which is distributed all over the world. This parasite can cause adverse health impacts on the immunodeficient individuals such as AIDS patients or organ transplant recipients (Xiao et al., 2010; Robert-Gangneux and Dardé, 2012; Chemoh et al., 2015). *T. gondii* can survive in nearly all nucleated cells (Blume et al., 2009; MacRae et al., 2012; Xia et al., 2019) and completes its intracellular reproduction inside a parasitophorous vacuole (PV) after invading the host cell. The synthesis or uptake of fatty acids and other molecules from the host cell is important for the growth and proliferation of intracellular pathogens. Fatty acids are the main components of the PV, the membranes of the apicoplast and other organelles in *T. gondii*, underscoring the significance of fatty acids for the survival of *T. gondii* (Mordue et al., 1999; Ramakrishnan et al., 2012; Botté et al., 2013).

The type II fatty acid biosynthesis pathway (FASII) in apicomplexan protozoa is located in a unique organelle known as apicoplast, which has an independent genome of prokaryotic origin with no counterpart in the human or animal host (Ralph et al., 2004), which makes apicoplast and FASII a source of novel drug targets for *T.* g*ondii* (Wiesner et al., 2008; Aygün and Mutlu, 2020). FASII, via a variety of enzymes, plays an important role in fatty acid metabolism. Phosphoenolpyruvate, after import to the apicoplast, is metabolized into pyruvate and converted into acetyl-CoA (a key precursor for FASII) via the action of pyruvate dehydrogenase complex (PDH) (Fleige et al., 2007). Acetyl-CoA can be also produced in the nucleus and cytosol via the cation of acetyl-CoA synthetase and ATP citrate lyase (Tymoshenko et al., 2015), and in the mitochondrion via the action of branched-chain α-keto acid dehydrogenase-complex (Oppenheim et al., 2014). Although cytosolic acetyl-CoA is required for the synthesis of parasite fatty acids, metabolic adaptation by the parasite compensates for any loss of mitochondrial acetyl-CoA (Kloehn et al., 2020).

The acyl carrier protein (ACP) plays a role in FASII. Deletion of ACP causes growth defect and decreases the virulence of *T. gondii* (Mazumdar et al., 2006). The synthesis of FASII in the apicoplast of *T. gondii* plays a key role in the survival of *T. gondii*, and the β-hydroxyacylacyl carrier protein dehydratase (FabZ; TGME49_321570) is essential for FASII metabolism in *T. gondii* (Wu et al., 2018; Krishnan et al., 2020). The protein coding genes *ODSCI* (TGGT1_269400) and *ODSCII* (TGGT1_313050) (ToxoDB database) belong to oxidoreductase, short-chain dehydrogenase/reductase family (Kowalik et al., 2009). According to KEGG analysis, *ODSCI* and *ODSCII* are involved in the FASII pathway. According to Enzyme Commission classification, they also belong to 3-oxoacyl-[acyl-carrier-protein] reductase, which is involved in type II fatty acid biosynthesis.

The knockout of *FabZ* led to the loss of apicoplast of *T. gondii* tachyzoites, and decreased the level of myristate (C14: 0) and palmitate (C16: 0), the synthetic products of FASII, which resulted in the inhibition of the growth of *T. gondii* (Krishnan et al., 2020). In the present study, we hypothesized that mutation in *T. gondii* genes *FabZ, ODSCI*, and *ODSCII* involved in fatty acid biosynthesis and metabolism may render the mutant parasite strains less virulent and incapable of establishing an infection. We constructed knockout strains using CRISPR-Cas9 technology, and evaluated the effects of deletion of *FabZ*, *ODSCI*, and *ODSCII* on the virulence of the mutant strains in mice and on the *in vitro* growth using plaque assay, intracellular proliferation assay and egress assay.

## Materials and Methods

### *Toxoplasma gondii* Cultures

*T. gondii* tachyzoites of the virulent RH (Genotype I) and less virulent PRU (Genotype II) strains were maintained *in vitro* in human foreskin fibroblasts (HFFs, ATCC^®^, SCRC-1041^TM^) as described previously (Zhang et al., 2021). The HFF cells were grown in Dulbecco’s Modified Eagle medium (DMEM) supplemented with 10% fetal bovine serum (FBS), 10 mM HEPES (pH 7.2), 100 Ug/ml streptomycin and 100 U/ml penicillin, and maintained at 37°C with 5% CO_2_.

### Construction of Knockout Strains

*FabZ*, *ODSCI*, and *ODSCII* knockout strains were constructed using CRISPR-Cas9 technology as described previously (Wang et al., 2020). Briefly, the single-guide RNA (sgRNA) of each gene was inserted into the template pSAG1-Cas9-SgUPRT plasmid using Q5 mutation kit (NEB) to replace the target RNA. The plasmid was sequenced and the positive plasmid was extracted using Endo-Free Plasmid DNA Mini Kit (Omega, Georgia, United States). The 5′ and 3′ homologous fragments of each gene were amplified from the genomic DNA of *T. gondii* RH strain using the designed 5′ and 3′ homologous arm specific primers. All the primers used in this study are listed in Supplementary Table 1. The DHFR resistant fragments were amplified using the pUPRT-DHFR-D plasmid as a template, and the three fragments (5′ and 3′ homologous arm and DHFR fragment) were cloned into the plasmid pUC19 employing the multi-fragment cloning approach using the CloneExpress II one-step cloning kit (Vazyme Biotech, Nanjing, China). The 5HR-DHFR-3HR fragment was amplified using the positive plasmid as a template to produce a homologous template, which was purified by agarose gel electrophoresis. About 40 μg of the CRISPR-Cas9 plasmid and 15 μg of the purified 5HR-DHFR-3HR fragment were co-transfected into freshly egressed *T. gondii* tachyzoites by electroporation. Finally, the clonal strain was obtained using a limiting dilution assay and 96-well plastic culture plates containing 3 μM pyrimethamine. The knockout strains were verified by PCR analysis. PCR 1 and PCR 3 were amplified from 5′ to 3′ of *FabZ*, *ODSCI*, and *ODSCII* by PCR1F/R and PCR3F/R, respectively, to verify whether the DHFR resistant fragment was successfully inserted into the corresponding knockout strain. PCR2F/R amplified product was about 700 bp in length, the knockout primers were designed at the upstream and downstream of *FabZ*, *ODSCI*, and *ODSCII* sgRNA-F to verify the successful knockout of the three genes (PCR2), and the wild-type (WT) strain was used as the control. The PCR reaction conditions were as follows: initial denaturation at 95°C for 4 min, followed by 35 amplification cycles (denaturation at 95°C for 30 s, annealing at 56°C for 30 s and extension at 72°C for 1 min), and a final extension step at 72°C for 10 min. The amplified products were examined on a 1.5% agarose gel with TAE buffer containing ethidium bromide.

### C-Terminal Tagging

The C-Terminal HA epitope tagging was used to establish the subcellular localization of *FabZ*, *ODSCI*, and *ODSCII*. CRISPR-Cas9 plasmids targeting the 3′ region of each gene were constructed as previously described (Cao et al., 2019). Using pLIC-3 × HA-DHFR plasmid as a template, PCR products containing about 1.5 kb (except stop codon) and 3 × HA and DHFR fragments at the 3′ of the target gene were amplified using specific primers and purified using agarose gel electrophoresis. The purified fragment and the constructed C-terminal (3′ region) specific CRISPR-Cas9 plasmid were electro-transfected into freshly egressed tachyzoites. To identify single positive clones and verify whether the HA fragment was successfully inserted into *T. gondii* tachyzoites, we used PCR and specific primers to amplify the *FabZ* gene. We also used immunofluorescence assay (IFA) to localize FabZ and acyl carrier proteins (ACP). Briefly, HFFs were infected with 1 × 10^6^ tachyzoites of RHΔ*FabZ* strain for 24 h. The cells were fixed with 4% paraformaldehyde (PFA) for 1 h, permealized for 30 min using 0.2% Triton X-100/phosphate-buffered saline (PBS), and blocked using 5% BSA for 30 min. Cells were incubated with primary antibodies (1:1,000), including mouse anti-SAG1 antibody to stain the tachyzoite’s plasma membrane or anti-ACP antibody to stain the apicoplast, and rabbit anti-HA antibody, overnight at 4°C; followed by incubation with the secondary antibody (Alexa Fluor 594 goat anti-mouse IgG;1:1,000) to stain SAG1 and ACP, or the secondary antibody (Alexa Fluor 488 anti-rabbit IgG; 1:1,000) to stain HA, for 1 h. Image analysis was performed using an TCS SP8 confocal scanning microscope (Leica, Germany).

### Immunofluorescence Localization of Apicoplast

HFFs were infected with 1 × 10^6^ tachyzoites of each of the knockout strains (RHΔ*FabZ*, RHΔ*ODSCI*, RHΔ*ODSCII*) and the WT RH strain and cultured at 37°C for 24 h. Subsequently, the cells were fixed with 4% paraformaldehyde (PFA) for 1 h, permealized for 30 min using 0.2% Triton X-100/PBS, and blocked using 5% bovine serum albumin (BSA) for 30 min. Cells were incubated with the primary antibodies (1:1,000), including rabbit anti-IMC1 (inner membrane complex) mouse antibody to stain the tachyzoites and anti-acyl carrier protein (ACP) antibody to stain the apicoplast, overnight at 4°C, followed by incubation with the secondary antibody (Alexa Fluor 488 anti-rabbit IgG; 1:1,000) to stain IMC1 or (Alexa Fluor 594 goat anti-mouse IgG;1:1,000) to stain the ACP 37°C for 1 h. Image analysis was performed using an TCS SP8 confocal scanning microscope (Leica, Germany).

### Plaque Assay

Approximately 500 tachyzoites of the three mutant RH strains and the WT strain were inoculated into 12-well culture plates containing HFF cell monolayers. Infected plates were incubated in 37°C and 5% CO_2_ for 7 days. After removing the culture medium, the infected cells were fixed with 4% PFA for 1 h and stained with 0.5% crystal violet solution for 30 min. The number and size of plaques were counted and analyzed as described previously (Wang et al., 2020).

### Intracellular Proliferation Assay

Tachyzoites (1 × 10^5^) of the three mutant strains and the WT strain were inoculated into cell culture dishes containing HFF cell monolayers. After 1 h, the medium was removed and the culture dishes were washed 3 times to remove any remaining extracellular tachyzoites. Fresh medium was then added to the infected cells and incubated at 37°C and 5% CO_2_ for 23 h. The cells were fixed with 4% PFA for 1 h, and then stained with mouse anti-SAG1 and Alexa Fluor 594 goat-anti mouse IgG. Parasitophorous vacuoles (PVs) were randomly examined under a fluorescence microscope, and the number of tachyzoites inside the PVs was determined.

### Egress Assay

Tachyzoites (1 × 10^5^) of the three mutant strains and the WT strain were inoculated into cell culture dishes containing HFF cell monolayers. After incubation at 37°C with 5% CO_2_ for 1 h, cultures were washed 3 times with PBS to remove any extracellular tachyzoites. Fresh medium was then added and culture was future incubated for 36 h, and infected cells were treated with 3 μM calcium ionophore A23187 diluted in DMEM as described previously (Zhang et al., 2021). Live cell microscopy was used to monitor and image the tachyzoite egress from the host cell.

### Quantitative Polymerase Chain Reaction (qPCR) Assay

Quantitative PCR (qPCR) was performed to quantify the expression of uracil phosphoribosyltransferase [(UPRT) (TGGT1_312480)] gene and apicoplast gene (TGGT1_302050) between WT strain and Δ*FabZ* strain. The 18 s rRNA gene was used as an internal reference for normalization. The qPCR assay was performed as described previously (Wu et al., 2009; Yeh and DeRisi, 2011; Reiff et al., 2012; Bansal et al., 2017). Genomic DNA was isolated from freshly egressed tachyzoites using Tiangen DNA extraction kit according to the manufacturer’s directions. The apicoplast and UPRT-specific gene sequences were amplified using Max Super-Fidelity DNA Polymerase (Vazyme). The amplification reaction mixture included 10 μl of 2 × SYBR Green *pro Taq HS* Premix (final concentration 1 ×) (Accurate Biology), 0.4 μl of 10 μM of each primer, 40 ng of the extracted *T. gondii* DNA, 0.4 μl of ROX Reference Dye, and sterile water to a final volume of 20 μl. qPCR was performed using an initial denaturation at 95°C for 30 s; followed by 40 cycles of amplification at 95°C for 10 s, 56°C for 20 s, and 72°C for 30 s.

### Tachyzoite-Bradyzoite Transformation

Tachyzoites of the knockout strains (PRUΔ*FabZ*, PRUΔ*ODSCI*, and PRUΔ*ODSCII*) and the WT PRU strain were used to infect the HFF cells cultured in alkaline RPMI-1640 medium (pH 8.2). After 3 days, the cells were fixed with 4% PFA for 1 h, subjected to permeabilization with 0.2% Triton X-100/PBS for 30 min at room temperature. Cells were blocked with PBS plus 5% BSA for 30 min and then incubated with the primary antibody (rabbit anti-IMC1) (1:1,000) overnight at 4°C, washed, and then incubated with goat anti-rabbit IgG Alexa Fluor 594 (1:1,000, Invitrogen, California, United States) 37°C for 1 h. Fluorescein isothiocyanate (FITC)-conjugated Dolichos biflorus lectin (DBL) was used to show the cyst wall (green). Slides were analyzed using a TCS SP8 confocal scanning microscope (Leica, Germany). We also quantified the rate of tachyzoite-bradyzoite transformation by subjecting *T. gondii-*infected HFF cells to immunostaining using DBL (a bradyzoite-specific marker) and IMC1 (stain of total parasites) as described previously (Xia et al., 2018). At least 100 PVs were examined in each parasite strain, and the experiment was repeated at least 3 independent times. The transformation rate of bradyzoites was calculated by dividing the number of DBL positive PVs by the number of IMC1 positive PVs.

### Characterization of RHΔ*FabZ*, RHΔ*ODSCI*, RHΔ*ODSCII* in Mice

Female specific-pathogen-free (SPF) Kunming mice (7-weeks-old) were purchased from the Center of Laboratory Animals, Lanzhou Veterinary Research Institute, Chinese Academy of Agricultural Sciences. Mice (8 mice/cage/group) were housed in SPF environment for 1 week in order to acclimatize to the experimental environment. Mice were housed in enriched conditions with 12-h light/dark cycle. The food and water were provided *ad libitum*. Mice were infected intraperitoneally (i.p.) with freshly egressed tachyzoites of the respective mutant strains or WT strains. The number of tachyzoites of RH or PRU strain used in the inoculation was 100 and 5 × 10^4^ per mouse, respectively. The level of disease severity in RH and PRU-infected mouse groups was carefully monitored twice daily. Well-defined humane endpoints were used to identify mice that must be euthanized to avoid unnecessary suffering. The number of cysts in the brain homogenates of the PRU-infected mice that survived until 30 days after infection were recorded as described previously (Wang et al., 2018).

### Bioinformatics Analysis

Information on the genomic characteristics of each gene, *FabZ*, *ODSCI*, and *ODSCII*, including the number of signal peptides, exons and transmembrane domains, and the temporal expression during the parasite cell cycle stages and in different *T. gondii* genotypes were obtained from ToxoDB.^1^

### Data Analysis

The data were analyzed by GraphPad Prism 7 (GraphPad Software, La Jolla, CA, United States). All the experiments were repeated 3 times and data are presented as means ± standard errors of the means (SEMs). The *F*-test was used for equal variance detection, and the means were compared using Student’s *t*-test or the Wilcoxon test.

## Results and Discussion

In this study we investigated whether deletion of *FabZ*, *ODSCI*, and *ODSCII* genes involved in the FASII pathway, using CRISPR-Cas9, influences the *in vitro* growth and *in vivo* virulence of *T. gondii*.

### Localization of *FabZ*, *ODSCI*, and *ODSCII*

**FIGURE 1:**
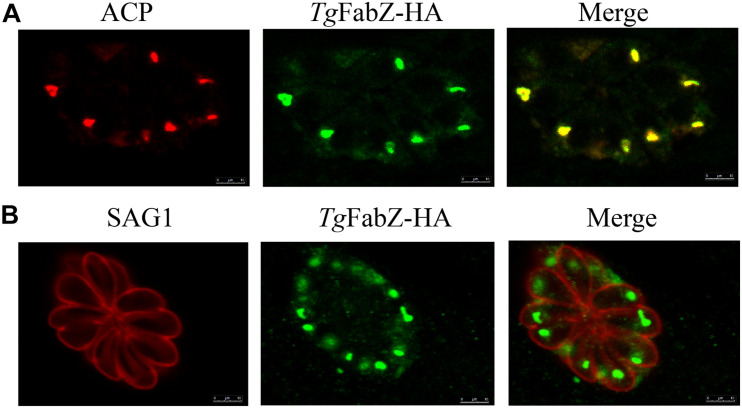
Colocalization of FabZ and apicoplast in *T. gondii* as determined by indirect immunofluorescence analysis. **(A)** Colocalization of acyl carrier protein (ACP) in the apicoplast (red) and *Tg*FabZ-HA (green). **(B)** Colocalization of the plasma membrane protein SAG1 of *T. gondii* tachyzoites (red) and *Tg*FabZ-HA (green). The results indicate that FabZ is located in the apicoplast. Scale bars, 10 μm.

The *FabZ*, *ODSCI*, and *ODSCII* were tagged with three HA epitopes at the C terminus to characterize their cellular localization. The results of IFA analysis showed that for FabZ, a fluorescence signal can be observed in the apicoplast, indicating that *Tg*-FabZ is expressed in the apicoplast in the WT strain (Figures 1A,B). In contrast, no fluorescence signal was detected in the apicoplast in case of *Tg*-ODSCI and *Tg*-ODSCII (data not shown), possibly due to their low expression in the tachyzoite stage.

### Construction of Δ*FabZ*, Δ*ODSCI*, and Δ*ODSCII* Strains

**FIGURE 2:**
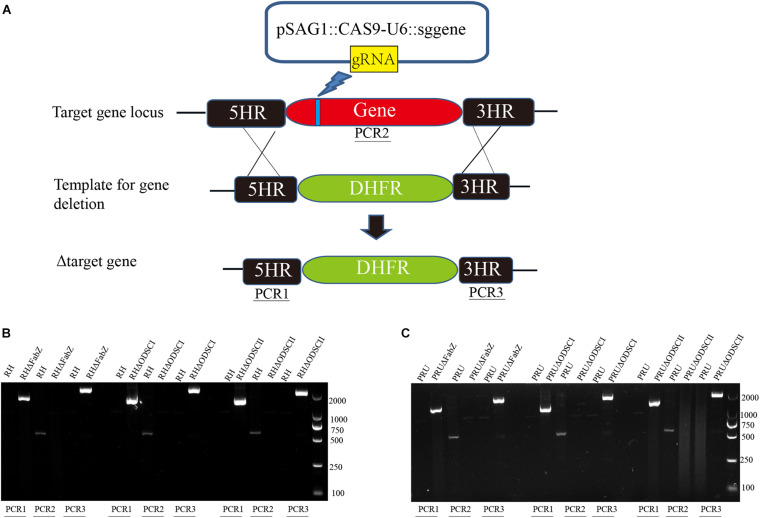
Construction of gene knockout strain using CRISPR-Cas9 system. **(A)** Schematic diagram of the CRISPR-Cas9 strategy used to knockout the target genes *FabZ*, *ODSCI*, and *ODSCII*. **(B,C)** The knockout strains were verified by PCR analysis. The results showed that PCR1 and PCR3 amplified bands in knockout strains, while RH and PRU wild strains had no bands, indicating that DHFR fragments were successfully inserted into knockout strains from 5′ to 3′ ends by homologous recombination. PCR2 showed that bands were amplified in RH and PRU wild-type strains, while knockout strains had no bands, showing that genes were successfully deleted.

To study the biological function of FabZ, ODSCI, and ODSCII in *T. gondii*, we deleted the corresponding encoding genes in RH and PRU strains using CRISPR-Cas9 system. The coding regions of *FabZ*, *ODSCI*, and *ODSCII* were replaced by 5HR-DHFR-3HR fragments using homologous recombination (Figure 2A). Then, the constructed Δ*FabZ*, Δ*ODSCI*, and Δ*ODSCII* strains were confirmed by PCR analysis. PCR1 and PCR3 were used to establish whether DHFR was successfully inserted into the target gene sequence. The PCR products were detected in the clonal mutant strains, but not in the parental RH or PRU strain. PCR2 was used to verify that the target gene is successfully deleted, where the amplified fragments were detected in the parental strain but not in the knockout strain (Figures 2B,C). These results showed that mutant strains, Δ*FabZ*, Δ*ODSCI*, and Δ*ODSCII*, were successfully constructed.

### The Effects on the *in vitro* Growth of *T. gondii*

**FIGURE 3:**
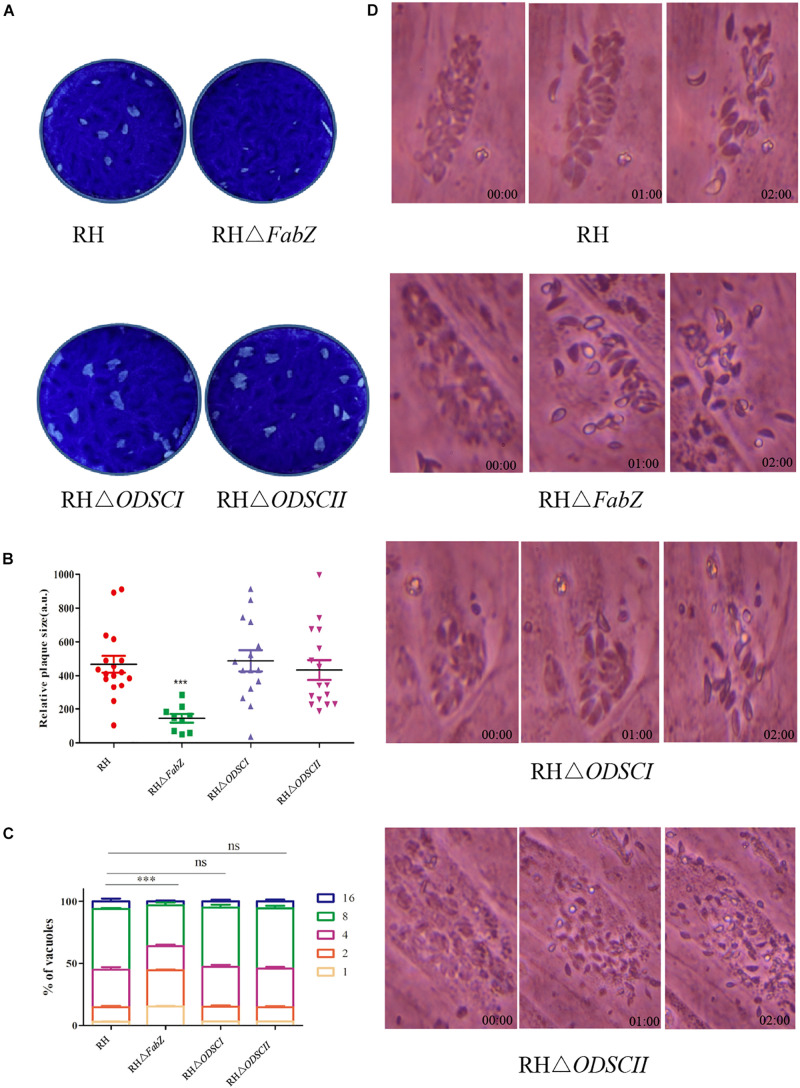
Characterization of the growth kinetics of the knockout strains *in vitro.* **(A)** Representative images of the plaque assay of HFFs infected with RH WT strain and mutant strains (RHΔ*FabZ*, RHΔ*ODSCI*, and RHΔ*ODSCII*) for 7 days. **(B)** Plaque’s size presented as arbitrary units (a. u.). The relative size of RHΔ*FabZ* plaque was significantly smaller and the number of plaque was fewer that of RH WT strain, and there was no significant difference between RHΔ*ODSCI* or RHΔ*ODSCII* and RH WT strain. **(C)** The replication of *T. gondii* in HFFs was quantified by counting the number of *T. gondii* tachyzoites (1, 2, 4, 8, 16) per vacuole 24 h after infection with RH WT strain, RHΔ*FabZ*, RHΔ*ODSCI*, or RHΔ*ODSCII* strain. RHΔ*FabZ* replication was significantly inhibited, while RHΔ*ODSCII* and RHΔ*ODSCII* strains had similar intracellular replication kinetics to WT strain. **(D)** HFFs were infected with RH WT strain, RHΔ*FabZ*, RHΔ*ODSCI*, or RHΔ*ODSCII* strain for 36 h, followed by treatment with 3 μM calcium ionophore A23187 to monitor the egress of tachyzoites at 0, 1, and 2 min after treatment. The results showed that the egressed pattern of WT strain was similar to that of all mutant strains. The data are presented as means ± SEMs (*n* = 3 in triplicates). ^∗∗∗^*P* < 0.001; ns, non-significant by Student’s *t*-test and Gehan-Breslow-Wilcoxon test.

In the present study, we determined whether FabZ, ODSCI, and ODSCII play a role in the growth of *T. gondii* by using the plaque assay, replication assay and egress assay. Significantly smaller and fewer plaques were formed by Δ*FabZ* strain compared to that produced by WT RH strain. There was no difference in the size and number of plaques formed by Δ*ODSCI* or ΔO*DSCII* when compared with WT RH strain (Figures 3A,B). These results show that lack of *FabZ* gene causes a considerable reduction in the growth of the RH strain; however, deletion of *ODSCI* and *ODSCII* did not have any impact on the rate of the parasite’s growth.

The results of intracellular proliferation assay showed that there were mainly 2 and 4 parasites in the PVs of Δ*FabZ* strain, and the number of PVs with 8 and 16 tachyzoites were significantly less than those detected in the PVs produced by the WT strain (Figure 3C), indicating that *FabZ* disruption significantly inhibited the replication of *T. gondii*. In contrast, *ODSCI* and *ODSCII* do not contribute to the parasite growth *in vitro* (Figure 3C). A similar finding was detected in *P. yoelii* where deletion of *FabZ*, a key enzyme in the FASII pathway, was found to prevent *P. yoelii* from forming merozoites during the liver infection stage (Vaughan et al., 2009).

We examined whether the deletion of *FabZ*, *ODSCI*, and *ODSCII* had an impact on *T. gondii* egress. The egress of *T. gondii* occurs when there are 64 or more tachyzoites inside the PV, and calcium is a key factor in mediating this process. 3 μM calcium ionophore A23187 was used to stimulate the tachyzoites release from the PV (Black et al., 2000a; Black and Boothroyd, 2000b; Arrizabalaga et al., 2004; Caldas et al., 2009). The results showed that most tachyzoites of Δ*Fab*Z, Δ*ODSCI*, or Δ*ODSCII* and WT strain egressed within 2 min, indicating that the knockout of these three genes do not have any impact on the egress of *T. gondii* (Figure 3D).

### Effect of *FabZ*, *ODSCI*, and *ODSCII* Deletion on Apicoplast of *T. gondii*

**FIGURE 4:**
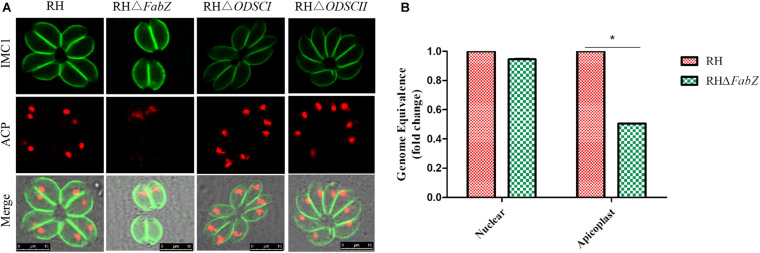
The effect of gene knockout on the apicoplast of *T. gondii* RH strain. **(A)** Immunofluorescence localization of the anti-acyl carrier protein (ACP) in the apicoplast using an apicoplast-specific antibody. The ACP specific fluorescence was hardly detected in the apicoplast of tachyzoites of Δ*FabZ*, but was clearly visible in the tachyzoites of Δ*ODSCI*, Δ*ODSCII*, and WT strains. Scale bars, 10 μm. **(B)** Quantitative PCR was performed to detect apicoplast and nuclear gene in Δ*FabZ* and WT RH strains. The expression of the apicoplast gene in Δ*FabZ* strain was significantly decreased. The data are presented as means ± SEMs (*n* = 3 in triplicates). *P < 0.05; ns, non-significant by Student’s *t*-test and Gehan-Breslow-Wilcoxon test.

The ACP fluorescent signal was hardly observed in the apicoplast of Δ*FabZ* tachyzoites, but was clearly detected in the tachyzoites of Δ*ODSCI* or Δ*ODSCII* and WT strains (Figure 4A). We further investigated the role of Δ*FabZ* in the stability of the apicoplast genome by comparing the replication of the apicoplast gene and the nuclear gene in Δ*FabZ* compared with WT by using qPCR. The results showed that compared with WT strain, the apicoplast gene expression of Δ*FabZ* was significantly decreased (Figure 4B), indicating that the presence of *FabZ* is essential for the maintenance of apicoplast genome of *T. gondii*.

In this study, the apicoplast gene (TGGT1_302050) and UPRT gene (TGGT1_312480) of *T. gondii* were successfully amplified. We also found that the apicoplast of Δ*FabZ* was lost, and the replications of the apicoplast genome, and the growth and intracellular proliferation were significantly reduced, indicating that FabZ plays a key role in the growth of *T. gondii*. These results show that the interruption of FASII pathway in the apicoplast by deleting *FabZ* may be the cause of the observed growth defects in Δ*FabZ* strain.

### Effect of Deletion of *FabZ*, *ODSCI*, and *ODSCII* on Phenotypic Transformation

**FIGURE 5:**
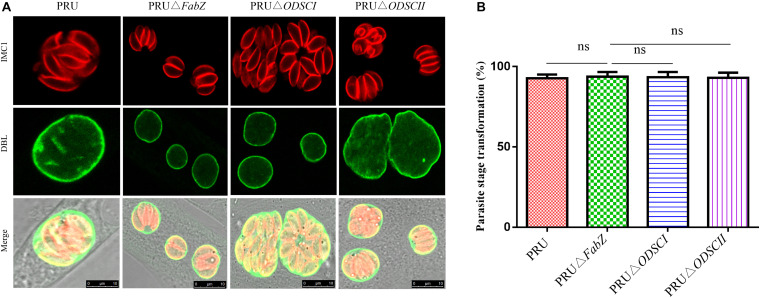
Tachyzoite-bradyzoite transformation of Δ*FabZ*, Δ*ODSCI*, or Δ*ODSCII* using immunofluorescence analysis. HFFs were infected with PRU WT, PRUΔ*FabZ*, PRUΔ*ODSCI*, or PRUΔ*ODSCII* strains and maintained in alkaline medium (pH = 8.2) for 3 days, then the parasites were stained with anti-IMC1 (inner membrane complex) and Alexa Fluor 594 (red). The parasite cyst walls were stained with FITC-conjugated Dolichos biflorus lectin, DBL (green). **(A)** The deletion of *FabZ*, *ODSCI*, or *ODSCII* in PRU strain did not have any remarkable impact on the tachyzoite-bradyzoite transformation ability. Scale bars, 10 μm. **(B)** No significant difference in the parasite stage transformation was detected between any of the tested parasite strains.

After incubating tachyzoites with RPMI-1640 (pH 8.2) for 3 days, the parasite cyst wall and tachyzoites were stained with DBL and IMC1, respectively. IFA results showed that Δ*FabZ*, Δ*ODSCI*, and Δ*ODSCII* were similar to WT PRU strain, all of the analyzed strains exhibit parasite DBL positive signal (green) (Figure 5A), suggesting that FabZ, ODSCI, and ODSCII do not contribute to the tachyzoite-bradyzoite transformation process. No significant difference was detected in the tachyzoite-bradyzoite transformation rate between any of the examined *T. gondii* WT and mutant PRU strains (Figure 5B).

### The Virulence of Δ*FabZ*, Δ*ODSCI*, and Δ*ODSCII* in Mice

**FIGURE 6:**
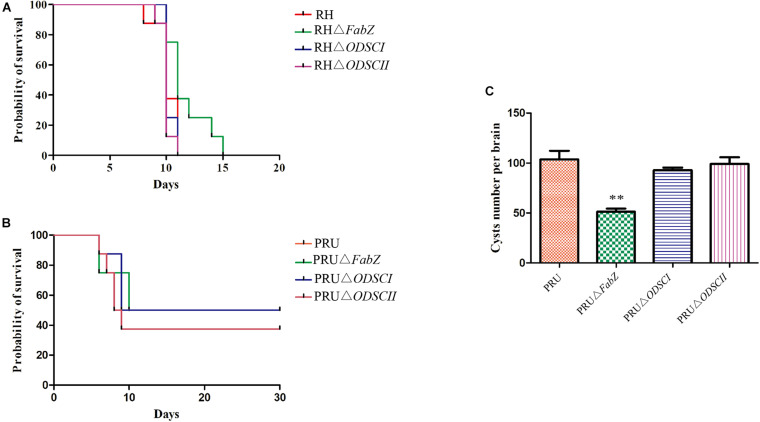
The virulence and cyst forming ability of Δ*FabZ*, Δ*ODSCI*, or Δ*ODSCII in vivo*. **(A)** The survival of Kunming mice inoculated by 100 tachyzoites of the RH (mutant or wild-type) strains. **(B)** The survival of Kunming mice infected by 5 × 10^4^ tachyzoites of the PRU (mutant and wild-type) strains. Mice infected by RH **(A)** or PRU **(B)** strain were checked twice daily by two independent observers for clinical signs and a Kaplan Meier survival curve plotted the infected mice that reached their humane endpoints. **(C)** The number of cysts in the mouse brain homogenates were counted at 30 days after mice were infected by 5 × 10^4^ PRU tachyzoites. The data are shown as means ± SEMs (8 mice/group). ^∗∗^*P* < 0.01; ns, non-significant by Student’s *t*-test.

We examined the impact of deletion of *FabZ*, *ODSCI*, and *ODSCII* on the pathogenicity of *T. gondii*. Approximately 100 tachyzoites of each strain, including RH, RHΔ*FabZ*, RHΔ*ODSCI*, and RHΔ*ODSCII*, were injected into Kunming mice by i.p. inoculation. The mice infected by RHΔ*FabZ* strain reached their humane endpoint up to 15 dpi, while mice infected by RHΔ*ODSCI*, RHΔ*ODSCII*, or WT strain reached their humane endpoint up to 11 dpi (Figure 6A). The survival rate of mice inoculated with wild-type PRU was 62.5%, and the survival rates of mice inoculated with knockout strains PRUΔ*FabZ*, PRUΔ*ODSCI*, and PRUΔ*ODSCII* were 50, 50, and 62.5%, respectively, indicating that *FabZ*, *ODSCI*, and *ODSCII* genes do not play any role in the virulence of *T. gondii* PRU strain in mice (Figure 6B).

We examined the effects of PRUΔ*FabZ*, PRUΔ*ODSCI*, and PRUΔ*ODSCII* on the cyst formation of *T. gondii*. Approximately 5 × 10^4^ tachyzoites of WT PRU and each of the three mutant strains were injected by the i.p. route into Kunming mice. After 30 days the number of cysts in the brain of the survived mice was counted. As shown in Figure 6C, the number of cysts in mice infected by PRUΔ*FabZ* (51.25 ± 3.198) was significantly (*P* < 0.01) lower than that in mice infected by WT (103.7 ± 8.686), while the number of cysts in mice infected with PRUΔ*ODSCI* and PRUΔO*DSCII* was not significantly different from that of the WT-infected mice.

Previous studies have shown that ACP is a key enzyme in the FASII pathway, and that deletion of the corresponding gene leads to defects in the growth of parasites *in vitro*, and increases the survival time of mice infected with mutant strain compared to mice infected by WT strain (Behnke et al., 2006; Mazumdar et al., 2006). Our results showed that deletion of *FabZ* induced defects in the growth of *T. gondii in vitro*, improved the survival of mice infected by the RH strain, and decreased the number of brain cysts of the PRU strain, indicating the important role that FabZ plays in the growth and virulence of *T. gondii*.

### Expression Profiles of *FabZ*, *ODSCI*, and *ODSCII*

Many genes exhibit temporal expression patterns during the replication of *T. gondii* tachyzoites, which is related to biological processes specific to the parasite development and replication (Behnke et al., 2006). Previous studies have found that some ROP genes, such as *ROP5*, *ROP16*, and *ROP17*, are cell cycle-dependent (Behnke et al., 2006). However, some ROPs do not show periodic expression characteristics, such as *ROP21*, *ROP27*, and *ROP28* (Jones et al., 2017).

**FIGURE 7:**
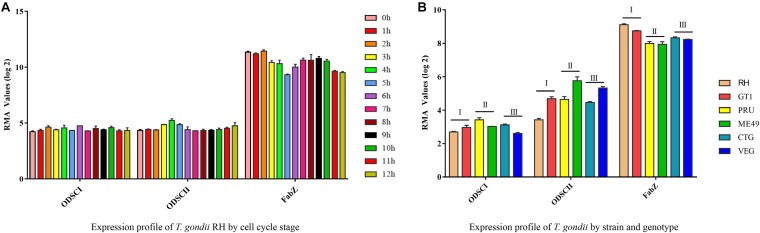
Expression profiles of *FabZ*, *ODSCI*, and *ODSCII* in *T. gondii*. **(A)** The temporal expression profile of *FabZ*, *ODSCI*, and *ODSCII* during the cell cycle of *T. gondii* RH strain. **(B)** The expression profile of *FabZ*, *ODSCI*, and *ODSCII* of *T. gondii* in type I (RH and GT1), type II (PRU and ME49) and type III (CTG and VEG) strains of *T. gondii*.

The expression profiles of *FabZ*, *ODSCI*, and *ODSCII* genes involved in FASII pathway were analyzed under different conditions according to the data available in ToxoDB. There was no difference in the expression of *FabZ* at different time points in the parasite’s respective cell cycle stages, but the expression of *FabZ* was significantly higher than that of *ODSCI* and *ODSCII* at the same time points (Figure 7A) (Behnke et al., 2010). Likewise, there was no difference in the expression of each gene between strains of the three main *T. gondii* genotypes (Type I, Type II, and Type III); however, the expression of *FabZ* was higher than that of *ODSCI* and *ODSCII* in the same examined strains (Figure 7B).

Studies have shown that the biological characteristics of some genes in *T. gondii* are expressed from a single exon and contain predicted signal peptides, and most proteins enter the secretory pathway mediated by signal peptides in eukaryotic cells (Devillers-Thiery et al., 1975; Lorenzi et al., 2016). The bioinformatics features, including the exon number, signal peptide and transmembrane domain of *FabZ*, *ODSCI*, and *ODSCII* are summarized in Table 1. Most of the proteins that perform biological functions in the apicoplast of *T. gondii* are encoded by the nuclear genome and transported to the apicoplast. Studies have shown that the signal peptide of the malaria parasite can transport proteins to the parasite’s apicoplast (Meyer et al., 2018). The present and previous studies detected specific signals of FabZ and ACP (Waller et al., 1998, 2000) in the apicoplast. These results suggest that *FabZ* and *ACP*, encoded by the nuclear genome of *T. gondii*, are transported to the apicoplast likely via signal peptides to exert their functions (Waller et al., 1998, 2000).

**TABLE 1:** Bioinformatic characteristics of *Toxoplasma gondii* genes.

Name	Gene ID	Product description	Exons	Phenotype value	TMHMM^a^	Predicted signal peptide
*FabZ*	TGME49_321570	Beta-hydroxyacyl-acyl carrier protein dehydratase	2	0.67	No	Yes
*ODSCI*	TGGT1_269400	Oxidoreductase, short chain dehydrogenase/reductase family protein	5	0.08	No	No
*ODSCII*	TGGT1_313050	Oxidoreductase, short chain dehydrogenase/reductase family protein	6	0.37	No	No

*^*a*^Prediction of transmembrane helices was performed using the TMHMM program version 2.0.*

## Conclusion

In this study, *FabZ*, *ODSCI*, and *ODSCII* involved in FASII pathway of the apicoplast were deleted using CRISPR-Cas9 system, and the *in vitro* growth and *in vivo* pathogenicity of the mutant strains were studied. Deletion of *FabZ* caused the loss of the apicoplast and reduced the growth of *T. gondii in vitro*, while deletion of *ODSCI* and *ODSCII* had no effect on the parasite’s growth. Deletion of *FabZ* reduced the virulence of the RH strain and decreased the brain cyst burden of the PRU strain in Kunming mice. Bioinformatics analysis showed that *FabZ* is likely to be transported to the apicoplast, via its signal peptides, to participate in the FASII pathway. These findings show that *FabZ* plays an important role in the pathogenesis of *T. gondii* infection.

## Data Availability Statement

The original contributions presented in the study are included in the article/Supplementary Material, further inquiries can be directed to the corresponding author/s.

## Ethics Statement

The animal study was reviewed and approved by the Animal Ethics Committee of Lanzhou Veterinary Research Institute, Chinese Academy of Agricultural Sciences.

## Author Contributions

X-QZ, M-XS, and HE conceived and designed this study, and critically revised the manuscript. X-PX performed the experiments, analyzed the data, and drafted the manuscript. W-GL, Z-WZ, L-XS, and Q-LL participated in the implementation of the study. All authors read and approved the final version of the manuscript.

## Conflict of Interest

The authors declare that the research was conducted in the absence of any commercial or financial relationships that could be construed as a potential conflict of interest.

## Publisher’s Note

All claims expressed in this article are solely those of the authors and do not necessarily represent those of their affiliated organizations, or those of the publisher, the editors and the reviewers. Any product that may be evaluated in this article, or claim that may be made by its manufacturer, is not guaranteed or endorsed by the publisher.

## Footnotes

1
http://toxodb.org

## References

[B1] Arrizabalaga G. Ruiz F. Moreno S. Boothroyd J. C. (2004). Ionophore-resistant mutant of *Toxoplasma gondii* reveals involvement of a sodium/hydrogen exchanger in calcium regulation. *J. Cell Biol.* 165 653–662. 10.1083/jcb.200309097 15173192 PMC2172388

[B2] Aygün C. Mutlu Ö. (2020). Computational characterisation of *Toxoplasma gondii* FabG (3-oxoacyl-[acyl-carrier-protein] reductase): a combined virtual screening and all-atom molecular dynamics simulation study. *J. Biomol. Struct. Dyn*. 52 1–18. 10.1080/07391102.2020.1834456 33063633

[B3] Bansal P. Tripathi A. Thakur V. Mohmmed A. Sharma P. (2017). Autophagy-related protein ATG18 regulates apicoplast biogenesis in apicomplexan parasites. *mBio* 8 e1468–e1417. 10.1128/mBio.01468-17 29089429 PMC5666157

[B4] Behnke M. S. Wootton J. C. Lehmann M. M. Radke J. B. Lucas O. Nawas J. (2006). Apicoplast fatty acid synthesis is essential for organelle biogenesis and parasite survival in *Toxoplasma gondii*. *Proc. Natl. Acad. Sci. U.S.A.* 103 13192–13197. 10.1073/pnas.0603391103 16920791 PMC1559775

[B5] Behnke M. S. Wootton J. C. Lehmann M. M. Radke J. B. Lucas O. Nawas J. (2010). Coordinated progression through two subtranscriptomes underlies the tachyzoite cycle of *Toxoplasma gondii*. *PLoS One* 5:e12354. 10.1371/journal.pone.0012354 20865045 PMC2928733

[B6] Black M. W. Arrizabalaga G. Boothroyd J. C. (2000a). Ionophore-resistant mutants of *Toxoplasma gondii* reveal host cell permeabilization as an early event in egress. *Mol. Cell. Biol.* 20 9399–9408. 10.1128/mcb.20.24.9399-9408.2000 11094090 PMC102196

[B7] Black M. W. Boothroyd J. C. (2000b). Lytic cycle of *Toxoplasma gondii*. *Microbiol. Mol. Biol. Rev.* 64 607–623. 10.1128/mmbr.64.3.607-623.2000 10974128 PMC99006

[B8] Blume M. Rodriguez-Contreras D. Landfear S. Fleige T. Soldati-Favre D. Lucius R. (2009). Host-derived glucose and its transporter in the obligate intracellular pathogen *Toxoplasma gondii* are dispensable by glutaminolysis. *Proc. Natl. Acad. Sci. U.S.A*. 106 12998–13003. 10.1073/pnas.0903831106 19617561 PMC2722337

[B9] Botté C. Y. Yamaryo-Botté Y. Rupasinghe T. W. Mullin K. A. MacRae J. I. Spurck T. P. (2013). Atypical lipid composition in the purified relict plastid (apicoplast) of malaria parasites. *Proc. Natl. Acad. Sci. U.S.A.* 110 7506–7511. 10.1073/pnas.1301251110 23589867 PMC3645554

[B10] Caldas L. A. de Souza W. Attias M. (2009). Microscopic analysis of calcium ionophore activated egress of *Toxoplasma gondii* from the host cell. *Vet. Parasitol.* 167 8–18. 10.1016/j.vetpar.2009.09.051 19875235

[B11] Cao X. Z. Wang J. L. Elsheikha H. M. Li T. T. Sun L. X. Liang Q. L. (2019). Characterization of the role of amylo-alpha-1,6-glucosidase protein in the infectivity of *Toxoplasma gondii*. *Front. Cell. Infect. Microbiol*. 9:418. 10.3389/fcimb.2019.00418 31867292 PMC6908810

[B12] Chemoh W. Sawangjaroen N. Siripaitoon P. Andiappan H. Hortiwakul T. Sermwittayawong N. (2015). *Toxoplasma gondii* - prevalence and risk factors in HIV-infected patients from Songklanagarind Hospital, southern Thailand. *Front. Microbiol*. 6:1304. 10.3389/fmicb.2015.01304 26635769 PMC4658439

[B13] Devillers-Thiery A. Kindt T. Scheele G. Blobel G. (1975). Homology in amino-terminal sequence of precursors to pancreatic secretory proteins. *Proc. Natl. Acad. Sci. U.S.A.* 72 5016–5020. 10.1073/pnas.72.12.5016 1061088 PMC388866

[B14] Fleige T. Fischer K. Ferguson D. J. Gross U. Bohne W. (2007). Carbohydrate metabolism in the *Toxoplasma gondii* apicoplast: localization of three glycolytic isoenzymes, the single pyruvate dehydrogenase complex, and a plastid phosphate translocator. *Eukaryot. Cell* 6 984–996. 10.1128/EC.00061-07 17449654 PMC1951530

[B15] Jones N. G. Wang Q. Sibley L. D. (2017). Secreted protein kinases regulate cyst burden during chronic toxoplasmosis. *Cell. Microbiol.* 19:10. 10.1111/cmi.12651 27450947 PMC5241228

[B16] Kloehn J. Oppenheim R. D. Siddiqui G. De Bock P.-J. Kumar Dogga S. Coute Y. (2020). Multi-omics analysis delineates the distinct functions of sub-cellular acetyl-CoA pools in *Toxoplasma gondii*. *BMC Biol*. 18:67. 10.1186/s12915-020-00791-7 32546260 PMC7296777

[B17] Kowalik D. Haller F. Adamski J. Moeller G. (2009). In search for function of two human orphan SDR enzymes: hydroxysteroid dehydrogenase like 2 (HSDL2) and short-chain dehydrogenase/reductase-orphan (SDR-O). *J. Steroid Biochem. Mol. Biol.* 117 117–124. 10.1016/j.jsbmb.2009.08.001 19703561

[B18] Krishnan A. Kloehn J. Lunghi M. Chiappino-Pepe A. Waldman B. S. Nicolas D. (2020). Functional and computational genomics reveal unprecedented flexibility in stage-specific *Toxoplasma* metabolism. *Cell Host Microbe* 27 290–306. 10.1016/j.chom.2020.01.002 31991093

[B19] Lorenzi H. Khan A. Behnke M. S. Namasivayam S. Swapna L. S. Hadjithomas M. (2016). Local admixture of amplified and diversified secreted pathogenesis determinants shapes mosaic *Toxoplasma gondii* genomes. *Nat. Commun.* 7:10147. 10.1038/ncomms10147 26738725 PMC4729833

[B20] MacRae J. I. Sheiner L. Nahid A. Tonkin C. Striepen B. McConville M. J. (2012). Mitochondrial metabolism of glucose and glutamine is required for intracellular growth of *Toxoplasma gondii*. *Cell Host Microbe* 12 682–692. 10.1016/j.chom.2012.09.013 23159057 PMC3990185

[B21] Mazumdar J. Wilson E. Masek K. Hunter C. A. Striepen B. (2006). Apicoplast fatty acid synthesis is essential for organelle biogenesis and parasite survival in *Toxoplasma gondii*. *Proc. Natl. Acad. Sci. U.S.A*. 103 13192–13197. 10.1073/pnas.0603391103 16920791 PMC1559775

[B22] Meyer C. Barniol L. Hiss J. A. Przyborski J. M. (2018). The N-terminal extension of the *P. falciparum* GBP130 signal peptide is irrelevant for signal sequence function. *Int. J. Med. Microbiol.* 308 3–12. 10.1016/j.ijmm.2017.07.003 28750796

[B23] Mordue D. G. Håkansson S. Niesman I. Sibley L. D. (1999). *Toxoplasma gondii* resides in a vacuole that avoids fusion with host cell endocytic and exocytic vesicular trafficking pathways. *Exp. Parasitol.* 92 87–99. 10.1006/expr.1999.4412 10366534

[B24] Oppenheim R. D. Creek D. J. Macrae J. I. Modrzynska K. K. Pino P. Limenitakis J. (2014). BCKDH: the missing link in apicomplexan mitochondrial metabolism is required for full virulence of *Toxoplasma gondii* and *Plasmodium berghei*. *PLoS Pathog*. 10:e1004263. 10.1371/journal.ppat.1004263 25032958 PMC4102578

[B25] Ralph S. A. van Dooren G. G. Waller R. F. Crawford M. J. Fraunholz M. J. Foth B. J. (2004). Tropical infectious diseases: metabolic maps and functions of the *Plasmodium falciparum* apicoplast. *Nat. Rev. Microbiol.* 2 203–216. 10.1038/nrmicro843 15083156

[B26] Ramakrishnan S. Docampo M. D. Macrae J. I. Pujol F. M. Brooks C. F. van Dooren G. G. (2012). Apicoplast and endoplasmic reticulum cooperate in fatty acid biosynthesis in apicomplexan parasite *Toxoplasma gondii*. *J. Biol. Chem.* 287 4957–4971. 10.1074/jbc.M111.310144 22179608 PMC3281623

[B27] Reiff S. B. Vaishnava S. Striepen B. (2012). The HU protein is important for apicoplast genome maintenance and inheritance in *Toxoplasma gondii*. *Eukaryot. Cell* 11 905–915. 10.1128/EC.00029-12 22611021 PMC3416497

[B28] Robert-Gangneux F. Dardé M. L. (2012). Epidemiology of and diagnostic strategies for toxoplasmosis. *Clin. Microbiol. Rev.* 25 264–296. 10.1128/CMR.05013-11 22491772 PMC3346298

[B29] Tymoshenko S. Oppenheim R. D. Agren R. Nielsen J. Soldati-Favre D. Hatzimanikatis V. (2015). Metabolic needs and capabilities of *Toxoplasma gondii* through combined computational and experimental analysis. *PLoS Comput. Biol*. 11:e1004261. 10.1371/journal.pcbi.1004261 26001086 PMC4441489

[B30] Vaughan A. M. O’Neill M. T. Tarun A. S. Camargo N. Phuong T. M. Aly A. S. (2009). Type II fatty acid synthesis is essential only for malaria parasite late liver stage development. *Cell. Microbiol.* 11 506–520. 10.1111/j.1462-5822.2008.01270 19068099 PMC2688669

[B31] Waller R. F. Keeling P. J. Donald R. G. Striepen B. Handman E. Lang-Unnasch N. (1998). Nuclear-encoded proteins target to the plastid in *Toxoplasma gondii* and *Plasmodium falciparum*. *Proc. Natl. Acad. Sci. U.S.A*. 95 12352–12357. 10.1073/pnas.95.21.12352 9770490 PMC22835

[B32] Waller R. F. Reed M. B. Cowman A. F. McFadden G. I. (2000). Protein trafficking to the plastid of *Plasmodium falciparum* is via the secretory pathway. *EMBO J*. 19 1794–1802. 10.1093/emboj/19.8.1794 10775264 PMC302007

[B33] Wang J. L. Bai M. J. Elsheikha H. M. Liang Q. L. Li T. T. Cao X. Z. (2020). Novel roles of dense granule protein 12 (GRA12) in *Toxoplasma gondii* infection. *FASEB. J*. 34 3165–3178. 10.1096/fj.201901416RR 31908049

[B34] Wang J. L. Li T. T. Elsheikha H. M. Chen K. Cong W. Yang W. B. (2018). Live attenuated PRU:Δ*cdpk2* strain of *Toxoplasma gondii* protects against acute, chronic, and congenital toxoplasmosis. *J. Infect. Dis*. 218 768–777. 10.1093/infdis/jiy211 29669003

[B35] Wiesner J. Reichenberg A. Heinrich S. Schlitzer M. Jomaa H. (2008). The plastid-like organelle of apicomplexan parasites as drug target. *Curr. Pharm. Des.* 14 855–871. 10.2174/138161208784041105 18473835

[B36] Wu L. Chen S. X. Jiang X. G. Cao J. P. (2009). *Toxoplasma gondii*: a simple real-time PCR assay to quantify the proliferation of the apicoplast. *Exp. Parasitol*. 123 384–387. 10.1016/j.exppara.2009.08.013 19720060

[B37] Wu L. Tang C. Wang J. Jin X. Jiang X. Chen S. (2018). Induction of FAS II Metabolic disorders to cause delayed death of *Toxoplasma gondii*. *J. Nanosci. Nanotechnol*. 18 8155–8159. 10.1166/jnn.2018.16396 30189932

[B38] Xia N. Yang J. Ye S. Zhang L. Zhou Y. Zhao J. (2018). Functional analysis of *Toxoplasma* lactate dehydrogenases suggests critical roles of lactate fermentation for parasite growth *in vivo*. *Cell. Microbiol*. 20:e12794. 10.1111/cmi.12794 29028143

[B39] Xia N. Ye S. Liang X. Chen P. Zhou Y. Fang R. (2019). Pyruvate homeostasis as a determinant of parasite growth and metabolic plasticity in *Toxoplasma gondii*. *mBio* 10:e00898. 10.1128/mBio.00898-19 31186321 PMC6561023

[B40] Xiao Y. Yin J. Jiang N. Xiang M. Hao L. Lu H. (2010). Seroepidemiology of human *Toxoplasma gondii* infection in China. *BMC Infect. Dis*. 10:4. 10.1186/1471-2334-10-4 20055991 PMC2818656

[B41] Yeh E. DeRisi J. L. (2011). Chemical rescue of malaria parasites lacking an apicoplast defines organelle function in blood-stage *Plasmodium falciparum*. *PLoS Biol*. 9:e1001138. 10.1371/journal.pbio.100113 PMC3166167 21912516

[B42] Zhang Z. W. Li T. T. Wang J. L. Liang Q. L. Zhang H. S. Sun L. X. (2021). Functional characterization of two thioredoxin proteins of *Toxoplasma gondii* using the CRISPR-Cas9 system. *Front. Vet. Sci*. 7:614759. 10.3389/fvets.2020.614759 33521087 PMC7841047

