# Supplementary material for: The Role of Type II Fatty Acid Synthesis Enzymes FabZ, ODSCI, and ODSCII in the Pathogenesis of Toxoplasma gondii Infection
Source: Front Microbiol. 2021 Aug 31;12:703059. doi: 10.3389/fmicb.2021.703059 (PMC8438308; doi:10.3389/fmicb.2021.703059)
Supplement: Supplementary file 1 [file Table_1.DOCX]

**TABLE S1** **| Primers used in this study**

| Primer name | Primer sequence (5′ → 3′) |
| --- | --- |
| SgFabZ | GTTCGAGCCGGGAAAGCGTGGTTTTAGAGCTAGAAATAGC |
| Sg3FabZ | TCGTACGATGAAGTTGTACAGTTTTAGAGCTAGAAATAGC |
| SgODSCI | GATGAAGGACGACAGTGGAAGTTTTAGAGCTAGAAATAGC |
| Sg3ODSCI | GACCACGAGGAACGCACCAAGTTTTAGAGCTAGAAATAGC |
| SgODSCII | GCAGGCAGCGAAGCAAGATGGTTTTAGAGCTAGAAATAGC |
| Sg3ODSCII  PCR-1R  PCR-3F | GAAAACAGCCACGAAAAAGCGTTTTAGAGCTAGAAATAGC  ATGCTAATTCCTTTCTACTTTGGC  TGACGCAGATGTGCGTGTATCCAC |
| FABZ-PCR1-F  FABZ-PCR3-R  FabZ-KOF | TGCTGGCTGTAGTCCAGACT  CCTACTTCAGGTCAGCTTTCC  AGCTAACCCCGTCTTTGATGT |
| FabZ-KOR | TCCACGAACGCCTTTCCAGTA |
| ODSCI-KOF | ATACGCAACGGTAGCGTCATG |
| ODSCI-KOR ODSCI-PCR1-F  ODSCI-PCR3-R | GGATAAAACGAAACCCAGCAA  AGAGGACAGTGCAATGCTGC  GTTCTTTCATCGGAAGACGGC |
| ODSCII-KOF | GCCAGGCGACAAGATAAACAA |
| ODSCII-KOR  ODSCII-PCR-1-F  ODSCII-PCR-3-R | CTAAAAGGCAAACTCCCACCA  ACGCAGTCTCTTGAAGGAAGCG  CTGCATGCACTTTGCAGACAC |
| U5FabZ-Gbison-F | GGTTTTCCCAGTCACGACGTTGATGCTGCTGATTGTAGTTTT |
| U5FabZ-Gbison-R | GGATTTACAGCCTGGCGAAGCTTCCTGGACCGACAGTAAATGCT |
| U3FabZ-Gbison-F | CTATGCACTTGCAGGATGAATTCGAATGCGGGGATAGGGTGAAT |
| U3FabZ-Gbison-R | GAGCGGATAACAATTTCACAACGACGGTCCGAGTGTTACGA |
| U5ODSCI-Gbison-F | GGTTTTCCCAGTCACGACGTTCAGGGAAAATTCTTGCGTCAC |
| U5ODSCI-Gbison-R | GGATTTACAGCCTGGCGAAGCTT AGAAACCCACAGAAGGCGAAG |
| U3ODSCI-Gbison-F | CTATGCACTTGCAGGATGAATTCGATTTCAGACGCCGTCAGTCA |
| U3ODSCI-GbisonR | GAGCGGATAACAATTTCACACAGGAGCCTTTACGTGTAGCC |
| U5ODSCII-Gbison-F | GGTTTTCCCAGTCACGACGTTAAGTTGTTCTCCACCTAACGG |
| U5ODSCII-Gbison-R | GGATTTACAGCCTGGCGAAGCTT AGATGAAGTTCAACGCACCAG |
| U3ODSCII-Gbison-F | CTATGCACTTGCAGGATGAATTCCTGACACGGTCGCCAGGTATA |
| U3ODSCII-GbisonR | GAGCGGATAACAATTTCACAAATGTAAGAAACGCCCACTCG |
| apicoplast-F  apicoplast-R  UPRT-F  UPRT-R  18SRNA-F  18SRNA-R | TCTATTGCAATGGAAAAAGGTATG  TCAATGGTAGAGCAAAGGACTG  ACTGCGACGACATACTGGAGAAC  AAGAAAACAAAGCGGAACAACAA  GCTGACTACGTCCCTGCCC  ACAATTCATCATATCTTTCAATCGGTA |
